# The Effect of Biochar on Tomato (*Solanum lycopersicum*) Cultivar Micro-Tom Grown under Continuous Light

**DOI:** 10.1007/s42729-024-02003-5

**Published:** 2024-11-06

**Authors:** Larissa Nicholas, Aisling Devine, Iain Robertson, Ian Mabbett

**Affiliations:** 1 Department of Chemistry, Faculty of Science and Engineering, Swansea University, Swansea, Wales SA2 8PP UK; 2 Department of Biosciences, Faculty of Science and Engineering, Swansea University, Swansea, Wales SA2 8PP UK; 3 Geography Department, Faculty of Science and Engineering, Swansea University, Swansea, Wales, SA2 8PP UK

**Keywords:** Continuous light stress, Biochar, Yield, Tomato, Soil

## Abstract

Continuous lighting (CL) has the potential to increase crop yield in greenhouse production. Tomato plants, however, when exposed to CL develop inter-vascular chlorosis, a leaf injury which causes a reduction in chlorophyll content and necrosis. The application of biochar can reduce physiological stress in plants, we examine if biochar also reduces necrosis in tomatoes when grown under CL. Faecal sludge biochar was applied to an acidic soil to examine plant growth and yield in Micro-Tom tomato plants grown under continuous light. We examined soil and plant growth properties of three soil application treatments: a control soil, biochar treatment (4%w/w) (Biochar), and a combined biochar (2% w/w) and fertilizer (2% w/w) treatment (Biochar + Fert). Faecal sludge biochar addition produced plant heights 216% greater than control and above ground biomass 583% greater than control. The biochar and fertilizer treatment group produced a 487% increase in leaf number compared to biochar. The combined biochar and fertilizer treatment produced a 398% increase in dried above ground biomass and a 177% increase in dried fruit yield compared with biochar. Plants in the biochar and fertilizer treatment group showed less visual evidence of continuous light induced leaf injury.Biochar addition did not limit continuous light induced leaf chlorosis whereas combined biochar and fertilizer treatment resulted in a significant reduction in leaf injury and death. Overall, the application of biochar and biochar and fertilizer combined increased crop yield.

**Supplementary Information:**

The online version contains supplementary material available at 10.1007/s42729-024-02003-5.

## Introduction

Continuous light is photoperiods of up to 24 h of supplemental light and has the potential to increase plant growth and fruit yield in greenhouse production (Lanoue et al. 2021; Shao et al. 2022; Velez-Ramirez et al. 2011). However, tomato plants exposed to continuous light develop inter-vascular chlorosis (D. A. Demers et al. 1998a; Lanoue et al. 2021), a leaf injury indicated by yellowing spots and a mottled appearance of the leaf which eventually leads to a reduction in leaf chlorophyll content and necrosis (Arthur et al. 1930; D. A. Demers et al. 1998a; Hillman 1956; Logendra et al. 1990). Despite being first discovered in the 1920s, the mechanisms of chlorosis from exposure to continuous lighting is still not fully understood. Several theories exist including an accumulation of carbohydrates in the leaves (D.-A. Demers et al. 1998b; Dorais et al. 1996; Velez-Ramirez et al. 2011), photooxidative damage to the leaf pigments (Murage and Masuda 1997a), phytochrome signaling (Velez-Ramirez et al. 2019), and unbalanced excitation in photosystem I (PSI) and photosystem II (PSII) (Velez-Ramirez et al. 2014).

Enhanced plant growth upon biochar addition to soil is caused by several different mechanisms with the liming effect of alkaline biochar addition being a key process in this enhanced growth (Jeffery et al. 2011). Biochar also increases cation exchange capacity (Glaser et al. 2002), which coupled with the large surface area of biochar, can limit nutrient leaching (Lehmann and Joseph 2012). Another advantage of biochar addition is the increase in the water holding capacity of soil (Herath et al. 2013) caused by the microporous nature of biochar aiding in physically retaining water in the soil (Novak et al. 2009).

Biochar has been shown to benefit the management of stress in plants under various abiotic conditions such as drought stress (Li and Tan 2021; Yildirim et al. 2021; Zhang et al. 2020), salt stress (Ababsa et al. 2023; Eghlima et al. 2023; Kul et al. 2021), high temperature stress(Wang et al. 2021) and heavy metal toxicity (Kamran et al. 2020; Khan et al. 2022). Oxidative stress through both drought and salt stress results in the production of reactive oxygen species (ROS) (Hasanuzzaman et al. 2021). Biochar has enhanced the defense system in plants under drought stress by modifying the ROS scavenging enzymes(Mansoor et al. 2021) and under salt stress the application of biochar reduced ROS in the plants (Farhangi-Abriz and Torabian 2018; Mehmood et al. 2020). In plants the detoxification of stress-induced ROS is regulated by antioxidant enzymes such as superoxide dismutase (SOD), catalase (CAT) and peroxidase (POD) (Sharma et al. 2022). In fenugreek grown under salinity stress conditions, the addition of deashed biochar significantly decreased the activities of POD, SOD and CAT (Huang et al. 2024). Biochar treatment has also been shown to reduce cadmium phytotoxicity, increase POD activity and enhance growth in wheat under drought stress (Abbas et al. 2018). Despite an abundance of research investigating the effect of biochar on numerous types of abiotic stresses, to date there has been no research conducted studying the effect of biochar addition on tomato plants grown under continuous light stress.

We hypothesize that the application of biochar will reduce plant stress under continuous light conditions. In this study we examine to what extent biochar reduces continuous light induced chlorosis in tomato plants by specifically examining fruit yield, plant height, above ground biomass and total number of leaves.

## Methods

A pot trial using tomato (*Solanum lycopersicum*. L) cultivar Micro-Tom as a crop species was conducted to evaluate the impact of faecal sludge biochar and continuous light conditions on plant growth parameters. Biochar properties and soil textural and chemical properties are reported in Supplementary information (Tables S1, S2 and S3). Detailed methods for determining the biochar properties were previously reported (Nicholas et al. 2022).

### Plant Materials, Growing Conditions, and Experimental Design

The study was conducted as a pot experiment in a controlled temperature and light room. Temperature was maintained at a constant 21.2 °C (± 0.2) and a constant 24-hour photoperiod was maintained at an intensity of 120 µmol m^− 2^. After 91 days day/night heating temperatures were set to 15 °C /21°C and the constant 24-hour photoperiod was altered to a 12 -hour photoperiod and the experiment concluded after 155 days (Figure S1). The experimental design was a completely randomized design with three treatments and a total of nine replications. The three treatments were: (i) soil (Control); (ii) soil with biochar (BC); (iii) soil with biochar and fertilizer (BC + Fert). Soil and treatments were prepared in cylindrical plastic pots 9 cm in diameter, and 8.7 cm in height. All pots were well-watered with equal volumes of deionised water.

### Treatments

All pots contained 136 g of air-dried soil. The control treatment consisted of just soil (Control), the biochar treatment consisted of biochar applied at 4% w/w (Biochar) and the combined biochar and fertilizer treatment consisted of 2% w/w biochar and 2% w/w fertilizer (Biochar + Fert). The fertilizer used was a commercial organic fertilizer *Miracle-Gro* Performance Organics Granular Plant Food (NPK 8-5-5).

### Statistical Analysis

The relationship between the biochar soil treatments and the specific plant responses were examined using generalized linear models (GLM) in R (R 3.6.3). Plant growth responses which were fresh fruit yield, plant height, number of leaves, and above ground biomass were examined against the three different treatments (control, biochar and combined biochar and fertilizer). A GLM (Generalized linear model) and a post-hoc pairwise test was applied to examine the significance across the different treatments using the emmeans package (Lenth et al. 2022), which was adjusted accordingly for the different model distributions. For all plant responses, the GLM was modelled to a gamma distribution due to the positive skewness displayed.

## Results and Discussion

**Fig. 1:**
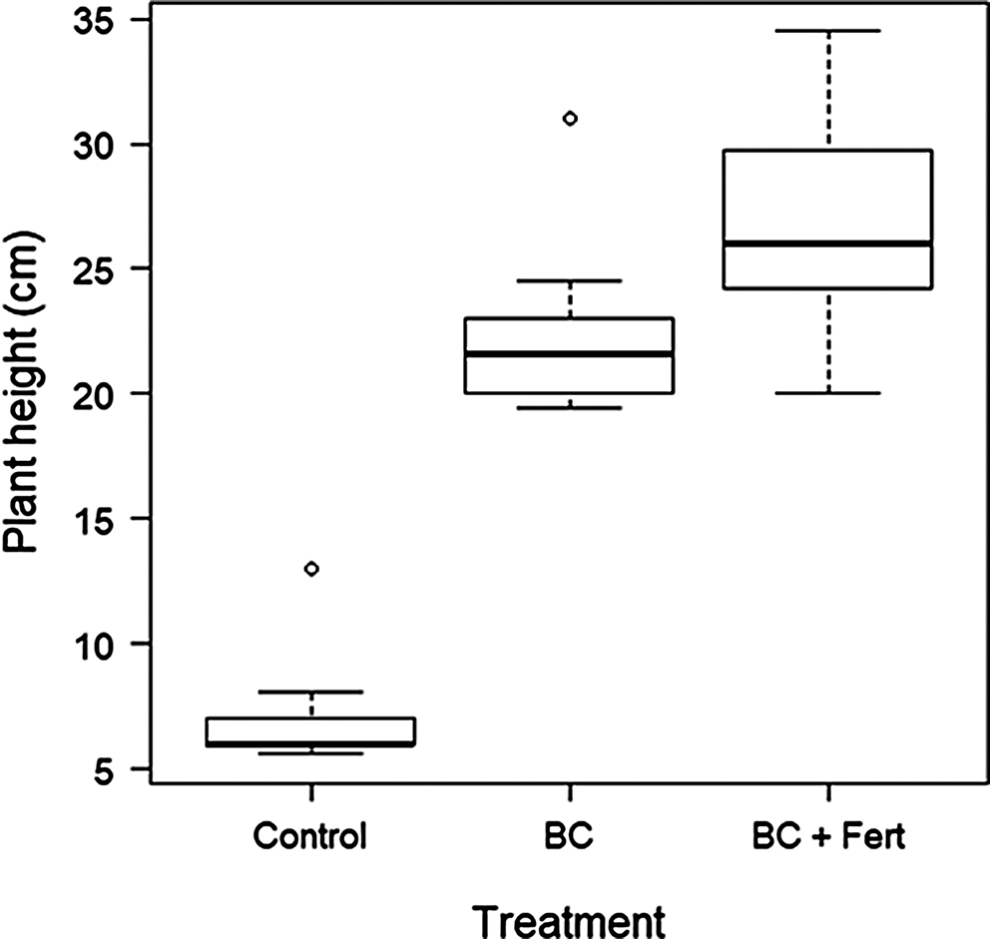
Plant height (cm) measured at harvest for each treatment, Control, BC (Biochar), and Biochar and fertilizer (BC + Fert). Box plots show minimum, first quartile, median (the horizontal solid line in the box), third quartile, and maximum. Open circle symbols indicate outliers

There was a significant difference between plant height for individuals that were treated with biochar and those that were not (Fig. 1; Table 1.). Biochar addition resulted in an increase in plant height of 216% compared to plant heights in the control group.

Plants in the BC + Fert group recorded a median plant height of 26.0 cm, an increase of 20% compared to plants in the BC treatment group with a median plant height of 21.6 cm. Both BC and BC + Fert were significantly different when compared to the control, recording increases in planet heights of 216% and 279% respectively but they were not significantly different from each other. (Table 1.).Nicholas et al. (2023) examined growth in Micro-toms, with biochar amendment under natural light conditions, which showed approximate total height of 10 cm, suggesting that continuous light had a significant impact on height accumulation, especially for plants subjected to biochar treatments and combined biochar and fertilizer treatments.

**Table 1:** The effects of biochar and fertilizer treatments on different plant growth responses using a gamma generalised linear model including pairwise comparison for plant height, number of leaves, above ground biomass and tomato fruit yield

Plant growth response	Treatment pairwise comparison		Estimate	Std.error	t value	*P*-value
Plant height	BC	BC + Fert	0.008	0.005	1.563	0.281
	BC	Control	-0.097	0.012	-8.137	< 0.001
	BC + Fert	Control	-0.104	0.012	-8.855	< 0.001
Above ground biomass	BC	BC + Fert	1.2	0.303	3.944	0.0018
	BC	Control	-8.73	2.048	-4.262	0.0008
	BC + Fert	Control	-9.93	2.028	-4.895	0.0002
Number of leaves	BC	BC + Fert	0.050	0.012	4.248	< 0.001
	BC	Control	-0.030	0.021	-1.428	0.344
	BC + Fert	Control	-0.080	0.017	-4.596	< 0.001
Tomato fruit yield	BC	BC + Fert	0.076	0.0178	4.262	0.0008
	BC	Control	-0.679	0.0979	-6.935	< 0.0001
	BC + Fert	Control	-0.755	0.0969	-7.794	< 0.0001

**Fig. 2:**
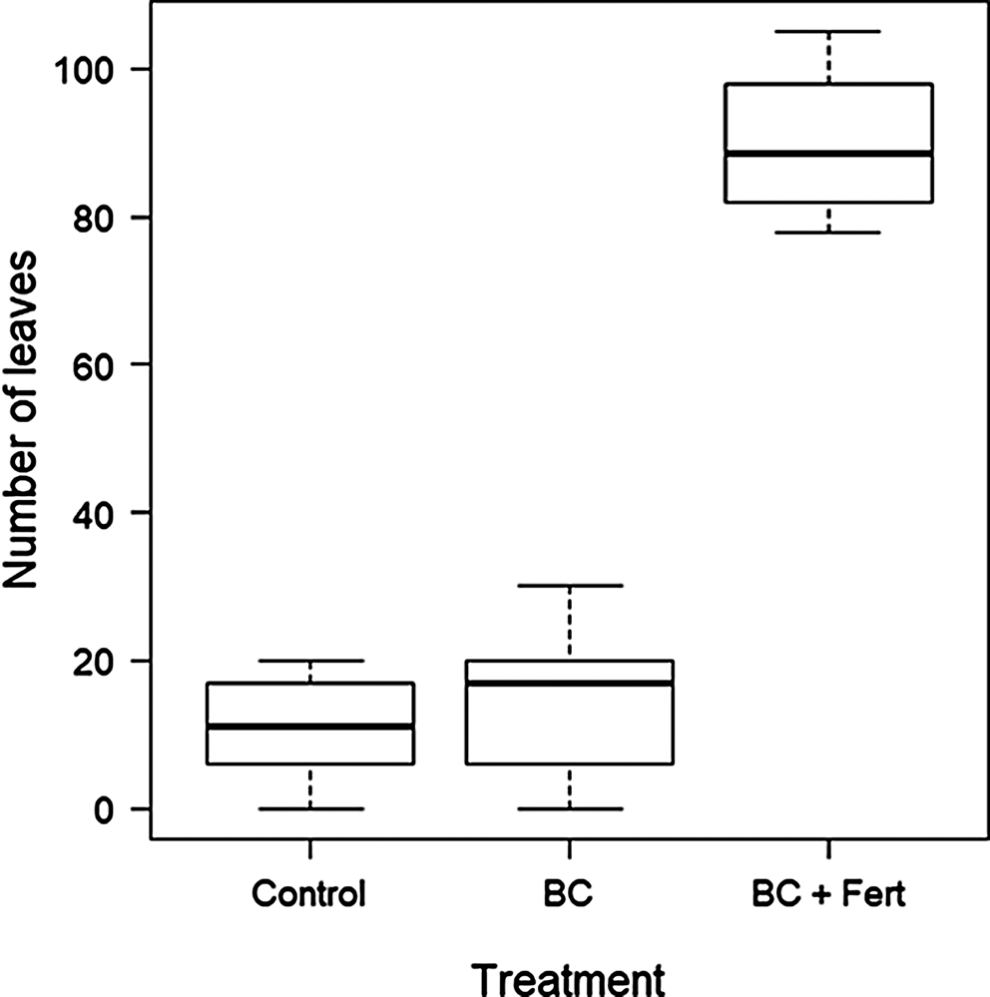
Number of leaves for each treatment, Control, BC (biochar), and BC + Fert (biochar and fertilizer. Box plots show minimum, first quartile, median (the solid line in the box), third quartile, and maximum

There was a marked significant increase (487%) in the median number of leaves with BC + Fert (89) compared to BC (17) (Fig. 2). There was no significant difference in leaf numbers between BC and control (Table 1.)

**Fig. 3:**
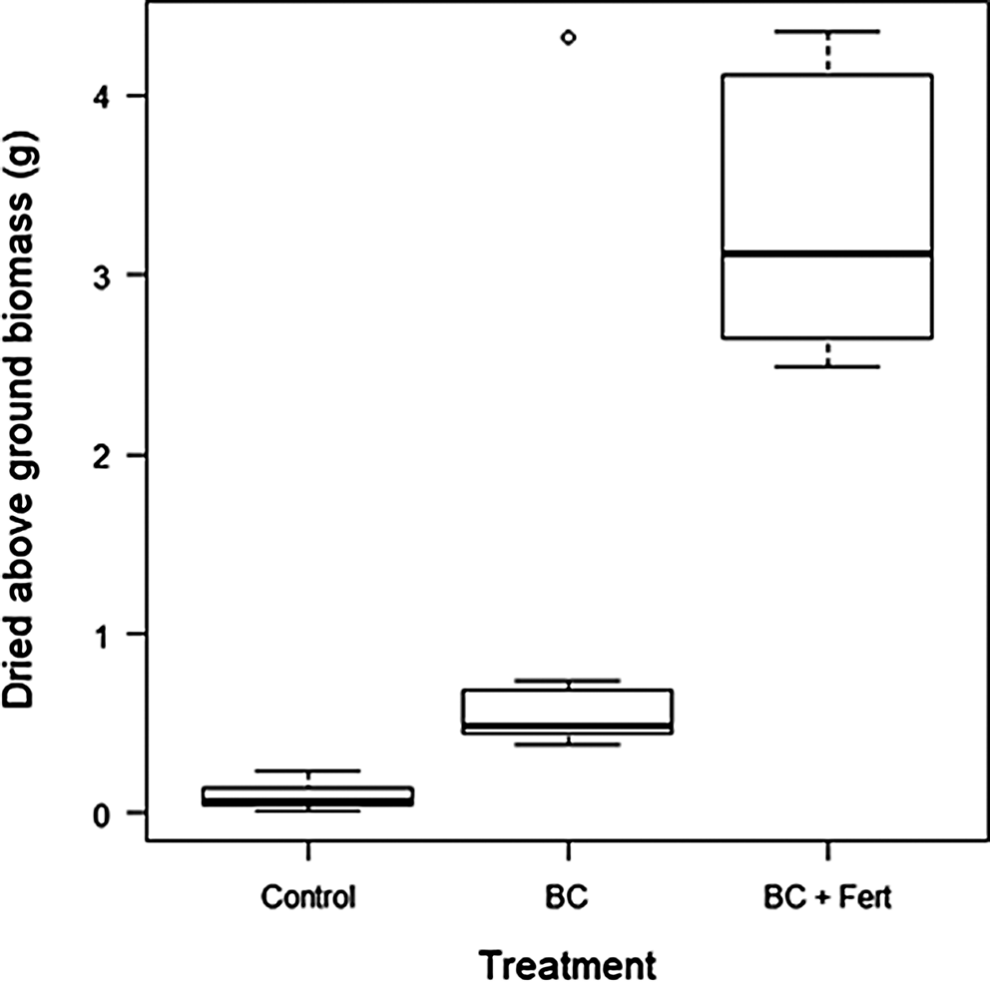
Above ground biomass (g) measured at harvest for each treatment, Control, biochar (BC), and biochar and fertilizer (BC + Fert). Box plots show minimum, first quartile, median (the solid line in the box), third quartile, and maximum. Open circle symbols indicate outliers

Above ground biomass for BC + Fert treatment (15.76 g) was significantly greater than BC (1.28 g) and control (0.14 g) (Fig. 3). Plants grown in the control treatment had markedly lower biomass than all other treatments and all combinations of the treatments were significant from each other (Table 1.).

The difference in above ground biomass compared to plant height is far more marked between BC + Fert and BC. Whilst there is a significant difference (*p* < 0.05) between all treatments, the above ground biomass for biochar only treated plants was much closer in value to that of the control, rather than the combined biochar and fertilizer treated plants. This corresponds with the visual observation of significant leaf loss in the biochar treated plants.

BC + Fert treatment produced a significant increase in the number of leaves of 487% and dried above ground biomass of 398% compared to BC (Figs. 2 and 3), which on its own had similar values to that of the control. This suggests that the biochar alone does not mitigate against continuous light-induced leaf injury and necrosis. The number of leaves produced by the control and the biochar treatments were not significantly different (Table 1), despite biochar amended plants outperforming the control group in other parameters such as plant height and fruit yield (Table 1), indicating leaf damage is still occurring under continuous light conditions.

**Fig. 4:**
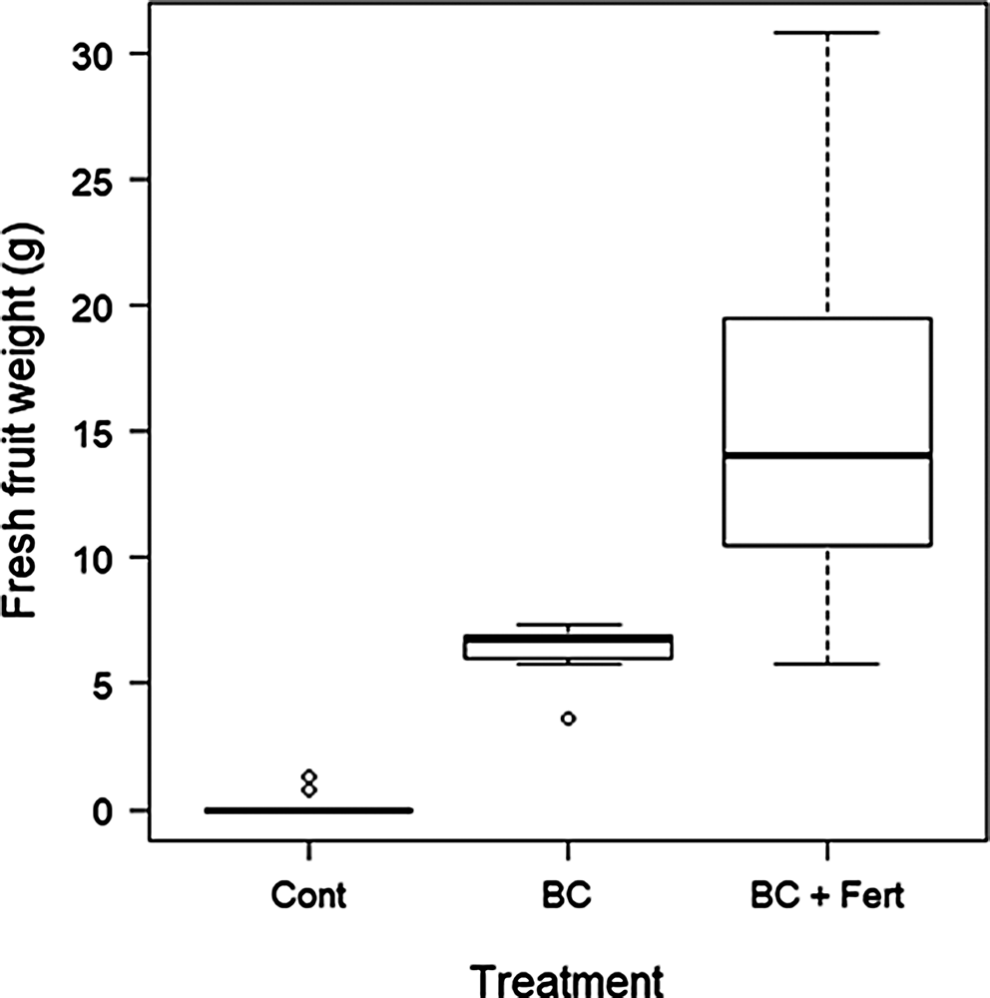
Tomato fruit yield measured at harvest for each treatment, Control, Biochar, and Biochar and fertilizer (Biochar + Fert). Box plots show minimum, first quartile, median (the solid line in the box), third quartile, and maximum. Open circle symbols indicate outliers

The application of biochar significantly increased fruit yield, all treatments were significantly different from each other with BC producing a 2,722% increase in dried fruit yield compared to control. However, BC + Fert produced the highest fruit yields overall (Table 1; Fig. 4.) Moreover, plants in the control treatment had exceedingly low yields under continuous light conditions, and the application of biochar alone appeared to mitigate against these low yields effectively. However, like in all other parameters of plant growth in this study the combination of biochar and fertilizer was shown to be the most effective under continuous light conditions.

The increased plant growth upon biochar addition is due to the liming effect as biochar increased soil pH (Figure S2). This liming effect of acidic soil increases the bioavailability of nutrients and adsorption (Bolan et al. 2023), with optimum phosphorus availability occurring at pH 5.5 -7.0. (Nigussie et al. 2012). Other mechanisms by which biochar can improve plant growth is the increased availability of nutrients within high ash content biochar(Smider and Singh 2014) and the increased CEC coupled with biochars larger surface area that limits nutrient leaching and improves nutrient retention (Song and Guo 2012). Biochar has also been shown to increase nitrifying bacterial abundance, microbial community structure and diversity(Zhang et al. 2017) and enhance the absorption of nutrients from the rhizosphere by improving the electrochemical properties of plant roots (Farhangi-Abriz and Ghassemi-Golezani 2023).

Separating the impact of biochar amendment on soil properties and the effect of continuous light on plant mechanisms is complex.

Under continuous lighting leaf chlorosis becomes evident and there is a synchronized increase in antioxidant enzymes superoxide dismutase (SOD), peroxidase (POD), and catalase (CAT) (Murage and Masuda 1997b). Biochar amendment has been shown to significantly decrease antioxidant enzymes SOD, CAT and POD activities in crops under salt stress due to an increase in soil porosity and a reduction in bulk density(Kul et al. 2021) and fluoride toxicity stress due to increasing soil pH and cation exchange capacity and (Ghassemi-Golezani and Farhangi-Abriz 2019).

Under salt stress, biochar addition reduced the content of the plant stress hormones (abscisic acid (ABA), 1-aminocyclopropane-1-carboxylic acid (ACC), jasmonic acid (JA), and salicylic acid (SA) in common bean seedlings (Farhangi-Abriz and Torabian 2018). Conversely, biochar addition increased the phytohormone indole-3-acetic acid (IAA) that regulates plant growth. A comprehensive study of over 1000 genes in biochar treated *Arabidopsis thaliana* and lettuce (*Lactuca sativa* L.) plants showed up-regulation in genes central for the promotion of growth seen in biochar addition (auxin and brassinosteroid). Crucially, down-regulation in genes related to plant immunity and defence (jasmonic and salicylic acid biosynthetic pathways) was also discovered with biochar addition (Viger et al. 2015).Tartaglia et al. (2020) also demonstrated that tomato plants treated with biochar showed a down-regulation of defence genes and up-regulation of a repressor gene of the JA signalling pathway.

The increased biomass and reduction in continuous light induced leaf necrosis in combined biochar and fertilizer treatment is possibly due to an increase in nutrients provided by the fertilizer rather than a reduction of the biochar. Previous work has shown an increase in fertilization has reduced the leaf injury and increased yield in mini cucumber plants (Hao et al. 2012). However, Demers et al. (1998a, b, c) concluded that leaf chloroses in tomato plants under continuous light were not associated with mineral nutrition problems. However, this does not appear to be the case in our study, where the application of combined biochar and fertilizer greatly improved growth and yield responses than just the application of biochar alone. Further research is needed to examine the physiological and mechanisms responses behind the role of light in nutrient absorption and plant growth and regulating plant stress (Xu et al. 2021).

## Conclusion

Biochar has been widely used as a soil amendment to improve plant responses to abiotic stress. This study is the first to examine the impact of biochar application on leaf chlorosis and necrosis of tomato plants under continuous light stress.

Biochar treatment without fertilizer was not as effective in mitigating against continuous light leaf damage and necrosis, compared to the combined biochar and fertilizer treatment suggesting that nutrient uptake plays a vital role in decreasing plant stress. It is possible that the application of biochar results in an up-regulation of growth genes concurrent with a down-regulation in plant defense genes and pathways. However, further experimental work is needed to understand the mechanisms behind the variation in continuous light induced leaf injury with different rates of biochar and fertilizer addition and to understand why the addition of biochar alone resulted in exacerbated leaf chlorosis and necrosis. Understanding the combined role of fertilizer and biochar to reduce plant stress has potential application to increase tomato crop yields at an industrial agricultural scale, if continuous or nearly continuous lighting conditions can be applied.

## Electronic Supplementary Material

Below is the link to the electronic supplementary material.

Supplementary Material 1

## Data Availability

The datasets generated and analyzed for this study are available upon request.
