# Supplementary material for: The Effect of Biochar on Tomato (Solanum lycopersicum) Cultivar Micro-Tom Grown under Continuous Light
Source: J Soil Sci Plant Nutr. 2024 Nov 6;24(4):6775–81. doi: 10.1007/s42729-024-02003-5 (PMC11666722; doi:10.1007/s42729-024-02003-5)
Supplement: Supplementary file 1 — Supplementary Material 1 [file 42729_2024_2003_MOESM1_ESM.docx]

Supplementary Information

**Journal of Soil Science and Plant Nutrition**

**The effect of biochar on tomato (*Solanum lycopersicum*) cultivar Micro-tom grown under continuous light**

Larissa Nicholas^1^*, Aisling Devine^2^, Iain Robertson^3^, Ian Mabbett^4^

^1^ Department of Chemistry, Faculty of Science and Engineering, Swansea University, Swansea, Wales, UK

^2^ Department of Biosciences, Faculty of Science and Engineering, Swansea University, Swansea, Wales, UK, a.p.devine@swansea.ac.uk

^3^ Department of Geography, Faculty of Science and Engineering, Swansea University, Swansea, Wales, UK, i.robertson@swansea.ac.uk

^4^ Department of Chemistry, Faculty of Science and Engineering, Swansea University, Swansea, Wales, UK, [i.mabbett@swansea.ac.uk](mailto:i.mabbett@swansea.ac.uk)

***** Correspondence: 804174@swansea.ac.uk

### Materials and Methods

The faecal feedstocks for the preparation of the biochars used in this study were sourced from three different faecal sludge and septage processors in India: Narsapur in Andhra Pradesh, Warangal in Telangana and Wai, Maharashtra. The sludge was pyrolyzed into biochar using a flame temperature operating range of 550-750°C. The process relies on autothermal operation, thus a limited supply of oxygen flows through an air fan into the main reaction chamber to allow for partial oxidation. The biochar was stored in an airtight box and quenched in a water bath. Three 5kg biochar samples were collected from each processor in September 2018. Previous work showed that there were some differences in biochar properties in terms of pH, pore volume, electrical conductivity, carbon content and ash content [50]. Therefore, the pot trial included three replicates of each biochar to determine the effect on plant height and fruit yield of each biochar. The basic characteristics of biochars WGL BC (Warangal biochar), NSP BC (Narsapur biochar), and WAI BC (Wai biochar) are given in Table S1.

**Supplementary Table S1.** Proximate analyses, elemental analyses, pH, EC and surface area measurements of biochars.

| Parameter | Unit | WAI BC | NSP BC | WGL BC |
| --- | --- | --- | --- | --- |
| pH | [] | 11.81 ± 0.01 | 11.82 ± 0.01 | 12.25 ± 0.01 |
| EC | [mScm^−1^] | 2.70 ± 0.09 | 1.79 ± 0.17 | 9.00 ± 0.02 |
| Moisture | [%] | 3.08 ± 0.01 | 2.15 ± 0.31 | 0.98 ± 0.05 |
| Ash | [%] | 62.3 ± 0.32 | 67.0 ± 2.68 | 88.3 ± 0.21 |
| C | [%] | 21.11 | 23.79 | 8.06 |
| N | [%] | 1.32 | 1.13 | 0.37 |
| H | [%] | 1.55 | 0.73 | 1.15 |
| S | [%] | 0.03 | 0.27 | 0.03 |
| O* | [%] | 13.7 | 7.1 | 2.1 |
| H/C | [] | 0.9 | 0.4 | 1.7 |
| C/N | [] | 18.7 | 24.6 | 25.4 |
| O/C | [] | 0.5 | 0.2 | 0.2 |
| SBET N_2_ | [m^2^g^−1^] | 3.52 ± 0.78 | 3.69 ± 0.36 | 12.07 ± 4.12 |
| N_2_ TPV | [cm^3^g^−1^] | 0.011 | 0.011 | 0.019 |
| SBET CO_2_ | [m^2^g^−1^] | 46.72 ± 7.0 | 74.20 ± 4.0 | 26.11 ± 2.6 |
| CEC | [cmol.kg^−1^] | 90.0 ± 6.5 | 41.9 ± 2.2 | 129.3 ± 2.3 |

(EC = Electrical Conductivity, C = Carbon, N = Nitrogen, S = Sulfur, Oxygen, SBET = Surface area measured by BET, TPV = Total pore volume, SSA = Specific Surface area, CEC = Cation Exchange Capacity)

*Oxygen was calculated from the subtraction of total percentage carbon, hydrogen, nitrogen, and sulfur and ash content from 100

The soil used for this study was collected from farmland at Cathelyd Isaf Farm (51°42’38.0” N; 3°54’40.9” W) near Swansea, Wales and has a loamy sand texture and is acidic in nature with a pH measured at 5.0 ± 0.06. A composite sample was collected down to 0.2 m of the topsoil layer, then sieved to < 5 mm . At the end of the experiment 5:1 deionised water to soil solutions were prepared and the pH of soil measured with a Voltcraft soil pH meter. Soil electrical conductivity was measured with a Whatman CDM 400 electrical conductivity meter.

**Supplementary Table 2**. Soil texture analysis carried out by Lancrop Laboratories

| **Analysis** | **Result** |
| --- | --- |
| Sand (%) | 84.65 |
| Silt (%) | 13.66 |
| Clay (%) | 1.69 |
| Texture Class | Loamy Sand |
| Very Coarse Sand (%) | 3.91 |
| Coarse Sand (%) | 19.54 |
| Medium Sand (%) | 25.65 |
| Fine Sand (%) | 24.4 |
| Very Fine Sand (%) | 11.15 |
| Stones >2mm (%) | 2.7 |

**Supplementary Table 3**. Soil chemical analysis carried out by Maria Santiso Taboada (University of Santiago de Compostela).

| Parameter | Units | Mean |
| --- | --- | --- |
| Nitrogen | % | 0.8 ± 0.01 |
| Carbon | % | 15.4 ± 0.04 |
| Available NH_4_^+^ | mgKg^-1^ | 13.8 ± 1.4 |
| Available NO_3_^-^ | mgKg^-1^ | 27.4 ± 0.8 |
| Available PO₄³⁻ | mgKg^-1^ | 16.9 ± 0.1 |
| Cation Exchange Capacity | Cmol/Kg^-1^ | 8.71 ± 0.38 |

Fertilizer

The fertilizer used was a readily available commercial organic fertilizer *Miracle-Gro* Performance Organics Granular Plant Food (NPK 8-5-5).

Growing conditions

Micro-tom tomato seeds were sown into trays using a commercial compost to encourage germination before being transplanted into the treatment pots. The seedlings were transferred to pots containing the soil treatments twenty-two days after sowing, when the first true leaves had appeared.

Specific Analyses

Plant heights were measured from soil to tip of the plant and commenced on day 34, once the plants had grown to a height that allowed accurate measurement. Tomatoes were harvested after 155 days and tomato yield from each plant recorded. The shoots and roots of each plant were divided into above ground and below ground biomass and oven-dried at 65 °C for 24 hours before weighing on a balance.

Figure 1. Humidity (%) and temperature (°C) in the controlled environment laboratory recorded via data logger for duration of continuous light experiment


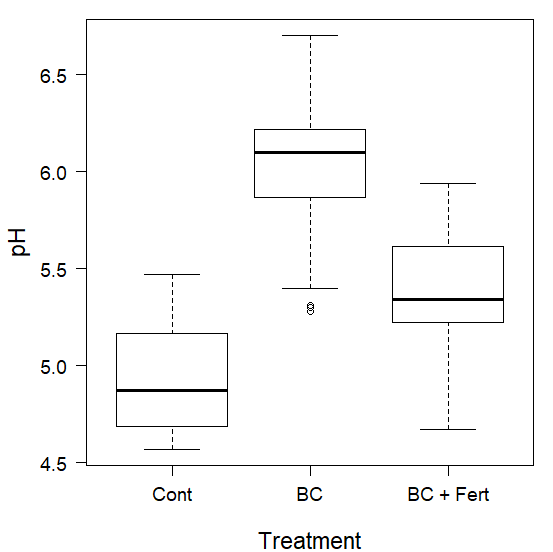


Figure 2**.** Soil pH measured at harvest for each treatment, Cont (control), BC (biochar), BC+Fert (biochar and fertilizer). Box plots show minimum, first quartile, median (the solid line in the box), third quartile, and maximum. Open circle symbols indicate outliers.
